# The Interlanguage Speech Intelligibility Benefit as Bias Toward Native-Language Phonology

**DOI:** 10.1177/2041669515613661

**Published:** 2015-11-16

**Authors:** Hongyan Wang, Vincent J. van Heuven

**Affiliations:** Leiden University Centre for Linguistics, The Netherlands; Department of English, Shenzhen University, China; Leiden University Centre for Linguistics, The Netherlands; Leiden Institute for Brain and Cognition, The Netherlands; Department of Applied Linguistics, University of Pannonia, Veszprém, Hungary

**Keywords:** Nonnative speech perception, interlanguage speech intelligibility benefit, matched interlanguage, non-matched interlanguage, linear modeling

## Abstract

Two hypotheses have been advanced in the recent literature with respect to the so-called *Interlanguage Speech Intelligibility Benefit* (ISIB): a nonnative speaker will be better understood by a another nonnative listener than a native speaker of the target language will be (a) only when the nonnatives share the same native language (matched interlanguage) or (b) even when the nonnatives have different mother tongues (non-matched interlanguage). Based on a survey of published experimental materials, the present article will demonstrate that both the restricted (a) and the generalized (b) hypotheses are false when the ISIB effect is evaluated in terms of absolute intelligibility scores. We will then propose a simple way to compute a relative measure for the ISIB (R-ISIB), which we claim is a more insightful way of evaluating the interlanguage benefit, and test the hypotheses in relative (R-ISIB) terms on the same literature data. We then find that our R-ISIB measure only supports the more restricted hypothesis (a) while rejecting the more general hypothesis (b). This finding shows that the native language shared by the interactants biases the listener toward interpreting sounds in terms of the phonology of the shared mother tongue.

## Introduction

Do non-native listeners understand foreign-accented English better than native English, especially when the non-natives share the same mother tongue? This paper is the first to rigorously quantify relevant data in a meta-analysis. It is commonly recognized that native speakers and native listeners outperform foreign speakers and listeners of the language. For instance, native (L1) listeners generally find fellow native talkers more intelligible than nonnative (L2) talkers, particularly in noisy conditions (Munro, 1998; Munro & Derwing, 1995). L1 speakers cause fewer word perception errors than foreign speakers of the language do. In a classical study, word recognition by native listeners for Serbian-, Japanese- and Punjabi-accented English was some 36% poorer than for native English speech in a range of signal-to-noise ratios and filtering conditions (Lane, 1967). More recently, it was shown that the word error rate of English spoken with a Mandarin accent was 11% against a mere 4% for native American control speakers, when in both cases the listeners were Americans (Munro & Derwing, 1995). Using a different methodology, native-speaker superiority was measured in terms of the speech reception threshold, that is, the noise level at which 50% of the words in the test materials are correctly recognized. Speech reception threshold was found to be at a 4-dB poorer signal-to-noise ratio when the Dutch listeners responded to Dutch speakers, than when the speakers were British learners of Dutch (Van Wijngaarden, 2001).

By the same token, L1 listeners have better scores, faster recognition times, and withstand more adverse listening conditions than L2 listeners do — at least when the test materials are recorded from fellow L1 speakers. Native listeners are better at recognizing degraded speech (telephone speech, synthetic speech, speech in noise) than nonnative speakers. For instance, Dutch listeners could recognize Dutch words from shorter onset portions than English learners of Dutch, even if the latter had resided in the Netherlands for 20 years or more (Nooteboom & Truin, 1980).

In the studies summarized earlier, information is exchanged between a native speaker and a native listener as the control condition and a native/nonnative pair of interactants for the experimental condition. In this comparison the native/nonnative pair is consistently outperformed by the native/native control pairs. Note that the comparison does not involve pairs of interactants who are both nonnative speakers of the language used. Somewhat surprisingly, it has been observed that nonnative speakers may be more intelligible than native speakers when the listener is also nonnative. Indeed, second-language learners often report that the speech of a fellow nonnative talker is easier to understand than the speech of a native talker. Bent and Bradlow (2003) advanced two hypotheses with respect to this phenomenon. The first hypothesis holds that a foreign talker of a language is more intelligible to any foreign listener of that language than a native speaker is. This is what Bent and Bradlow call the non-matched (or “mixed”) interlanguage speech intelligibility benefit (ISIB). Early evidence in support of this hypothesis has been provided by Nash (1969). The second, more restricted, hypothesis predicts that a foreign talker will be more intelligible to a foreign listener (than a native talker would be) only if the foreign talker and listener share the same mother tongue. This is what Bent and Bradlow call the matched (or “shared”) interlanguage benefit.

The theoretical underpinning of the unrestricted hypothesis seems tenuous. It has been observed that nonnative talkers speak rather slowly and hesitantly, which would benefit anyone who would have problems with decoding the message (e.g., Derwing & Munro, 2001). The slow speed of delivery and the insertion of pauses when the speaker is looking for words would allow the nonnative listener time to integrate what has been heard and to predict upcoming words. The beneficial effect of insertion of pauses (with compensation for slower rate of delivery) has been demonstrated for low-quality Dutch speech synthesis and for natural Dutch speech in noise (Scharpff, 1994; Scharpff & Van Heuven, 1988; Van Heuven & Scharpff, 1991), as well as for Danish perceived by Swedish listeners (Gooskens & Van Bezooijen, 2014). Moreover, the foreign talker will use a fairly restricted vocabulary comprising high-frequency words only (e.g., Cervatiuc, 2008; Milton & Meara, 1995) so that the listeners will not often be confronted with unfamiliar words. The benefit will probably disappear, we would argue, if the test materials were produced by a native speaker of the target language and manipulated such that the words and sentence structures (after minimal correction) and the gross temporal organization (speed of delivery as well location and length of pauses) would be the same as that used by the nonnative talker. We are not aware of any such study, however, so that our objection remains speculative.

Arguments supporting this more restricted hypothesis have been provided by many studies, for example, Smith and Rafiqzad (1979), Van Wijngaarden (2001), Van Wijngaarden, Steeneken, and Houtgast (2002), Imai, Flege and Walley (2003), Wang and Van Heuven (2003, 2004, 2006), and Wang (2007). Native speakers have a vast knowledge of the statistical regularities at all linguistic levels (sounds, syllables, morphemes, words, and sentences) and skillfully use any redundancy that may exist in the native language system (Cutler, 2012). These skills are much less developed in nonnative listeners. The sound categories of the target language are less well defined in the perceptual representation of nonnatives, and transitional probabilities that allow the native listener to predict upcoming sounds (or restore sounds that were missed) are not known to (let alone used by) the nonnative listener. This does not only apply to nonnative listeners who have learned the foreign language as adults, but it has been shown that even the sound categories in a second language that was acquired before the age of four (i.e., by so-called *early bilinguals*) are less well defined than for monolingual listeners (Sebastian-Galles & Soto Faraco, 1999).

Bent and Bradlow (2003) tested both hypotheses in one integrated experiment and claim that they found evidence in support of both. They point out that specific combinations of foreign speaker and listener language backgrounds yield better intelligibility scores than combinations involving a native speaker or listener, both when language backgrounds of the foreign speakers and listeners are mixed and when they are shared. However, the authors do not quantify the effect in a way that allows the reader to determine the magnitude of the interlanguage benefit, nor to check whether the benefit is larger for the shared interlanguage than for the mixed interlanguage situation. We would argue that this is an undesirable state of affairs. In fact, it is our contention that in none of the studies we are aware of (see below) has the ISIB been quantified and tested in an adequate way. More specifically we criticize the implicit choice in the literature to examine the ISIB in terms of absolute scores obtained by groups of listeners. This is a statistically inadequate and naïve way of testing the ISIB. Rather, we will argue that the ISIB should be evaluated in a relative manner, using insights from linear modeling, such that the relative ISIB (R-ISIB) is defined as the magnitude of the statistical interaction term that is needed on top of the main effects of speaker and listener language in order to make an error-free prediction of the absolute scores. We will demonstrate the feasibility and superiority of our approach by making a systematic comparison of ISIB and R-ISIB values in a reanalysis of a number of published studies (in fact, all published studies that exist and speak to the issue).

We will briefly introduce the basic idea behind linear modeling and explain the difference between main effects and interactions. We will illustrate the (simple) computational method to express the magnitude of the (shared or mixed) interlanguage benefit and then reanalyze the results of a number of earlier studies on these phenomena. This meta-analytic exercise will show, first of all, that the proposed relative measure of the interlanguage benefit yields the predicted effects (much more clearly so than when some absolute measure of the benefit is applied), and that the benefit is indeed larger when speakers and listeners have a shared native language between them than when the interactants have different native languages. Moreover, the meta-analysis will reveal that the mutual intelligibility is poorest when one of the interlocutors (whether speaker or listener) is native and the other is nonnative. We will call this the case of the native-speaker handicap.

## A Relative (R-ISIB)

Linear modeling is a statistical technique that allows a researcher to decompose the relationship between stimulus properties and response scores into main effects and interactions between main effects (e.g., Philips, 1999, pp. 131–142; Yamane, 1973, pp. 865–874).

Table 1 presents data taken from a study described in more detail by Wang (2007), Van Heuven and Wang (2007), and Wang and Van Heuven (2014) — see also Meta-Analysis: Testing the ISIB Hypotheses on Aggregated Data section. In an experiment, one representative male and one female speaker were selected from a larger peer group comprising 10 male and 10 female speakers for each of three native language backgrounds, that is, Mandarin-Chinese, Dutch, and American English. These speakers had produced the vowels of English in a fixed/hVd/context, which were then offered for forced choice identification to groups of Chinese, Dutch, and American English listeners (*N* = 36 for each listener group). Per speaker group the one male and female speaker had been selected with the shortest Euclidean distance from the peer group centroid in a two-dimensional plane defined by correct vowel identification and correct consonant identification by listeners in an earlier experiment who shared the language background of the speakers (shared interlanguage).

**Table 1.** Correct Vowel Identification Scores (%) Obtained by Three Groups of Speakers (Chinese, Dutch, American) Based on the Responses of Three Groups of Listeners (Chinese, Dutch, American).

Speakers	Listeners									
	(a) Raw (absolute) scores					(b) Deviations from grand mean				
	Ch.	Du.	Am.	${\bar{\text{x}}}_{\text{i}}$	${\bar{\text{x}}}_{\text{i}}-\bar{\text{x}}$	Ch.	Du.	Am.	${\bar{\text{x}}}_{\text{i}}$	${\bar{\text{x}}}_{\text{i}}-\bar{\text{x}}$
Chinese	300	400	450	39	−100	−100	−190	−90	−100	−100
Dutch	340	590	610	52	+30	+30	−150	+100	+120	+30
American	340	590	750	56	+70	+70	−150	+100	+260	+70
${\bar{\text{y}}}_{\text{j}}$	330	530	610	$\bar{\text{x}}=\bar{\text{y}}=49$	Σ = 0	Σ = 0	−160	+40	+120	Σ = 0
${\bar{\text{y}}}_{\text{j}}-\bar{\text{y}}$	−160	+40	+100	Σ = 0						

The linear model decomposes the scores in each of the nine cells (as listed in part of Table 1) in terms of their deviation from the grand mean, both in the speaker dimension and in the listener dimension (part B). The grand mean of the nine scores equals 49. Subtracting the grand mean from all cell scores does not affect any of the differences between cell scores, it just adjusts the grand mean to zero. In linear modeling, the cell scores are then represented as the arithmetic addition of three components: (a) the main effect of speaker group, that is, the deviation from ¯x_i_ + (b) the main effect of listener group, that is, the deviation from ¯y_j_ + , (c) the interaction between speaker and listener group, that is, whatever adjustment is needed to make the addition of all three components match the cell contents. The interaction term for each cell is what is needed to bridge the gap between the cell score that is expected on the basis of the linear addition of the main effects of speaker group and listener group (the “expected” score) and the actual “observed” score for that cell. We argue that the interaction component, that is, the difference Δ between the expected and observed score for a cell in Table 1 is the magnitude of R-ISIB.

Table 2 rearranges the scores in Table 1 in a format that is better suited to illustrate how the components of the linear model are obtained. The observed scores (column marked “Obs.”) are the mean percent correct vowel identification scores for each of the nine combinations of speaker and listener language backgrounds, copied from Table 1. In absolute terms, the best intelligibility scores are obtained when both speakers and listeners are native (75% correct vowel identification). It is not the case, however, that native-nonnative speaker-listener combinations yield consistently poorer intelligibility scores than pairs exclusively involving nonnative interactants — in contradistinction to what the interlanguage intelligibility benefit hypothesis predicts. In fact, the poorest results are obtained when both speakers and listeners are Chinese (30%), and the best result is found for the combination of Dutch listeners to American speakers (61%). Nor is it the case that nonnative speaker-listener combinations that share the same language between them (30% for Chinese–Chinese and 59% for Dutch–Dutch) yield consistently better scores than mixed nonnative combinations (34% and 40% for Chinese–Dutch and Dutch–Chinese, respectively). Clearly, then, testing the ISIB hypothesis in absolute terms fails miserably.

**Table 2.** Expected Vowel Identification Scores (% Correct) on the Basis of Grand Mean (= 49%) and Main Effects for Listener and Speaker L1.

	Interlocutor’s role						Exp.	Obs.	Δ
	Listener			Speaker					
	Native language	Mean	Effect	Native language	Mean	Effect			
1.	Chinese	33	−16	Chinese	39	−10	22	30	**+8**
2.	Chinese	33	−16	Dutch	52	+3	35	34	−1
3.	Chinese	33	−16	Am. English	56	+7	40	34	−6
4.	Dutch	53	+4	Chinese	39	−10	42	40	−2
5.	Dutch	53	+4	Dutch	52	+3	55	59	**+4**
6.	Dutch	53	+4	Am. English	56	+7	60	59	−1
7.	Am. English	61	+12	Chinese	39	−10	50	45	−5
8.	Am. English	61	+12	Dutch	52	+3	63	61	−2
9.	Am. English	61	+12	Am. English	56	+7	68	75	**+7**
	Grand mean	0			0		49	49	0

*Note.* Means for listener groups and speaker groups are specified, as well as the effect of group membership (i.e., group mean–grand mean). The expected scores (Exp.) are found by subtracting the effects of listener group and speaker group from the grand mean. Observed scores (Obs.) and residuals (Δ) are indicated. Shaded rows contain speaker–listener combinations with a shared interlanguage. Bolded delta’s represent the shared interlanguage (or native language) benefit. All percentages have been rounded off to the nearest integer.

Now let us look at these results in rather more relative terms. I argue that the 30% correct vowel identification obtained by the Chinese-Chinese speaker-listener combination, although the lowest score of all in absolute terms, is in fact much better than should be expected in comparison with the other scores. Table 2 shows that the 30% vowel identification score is better than what would be expected on the basis of the linear addition of the main effects of speaker group and listener group. The difference is, in fact, 8 points, that is, a R-ISIB value of 8.

The computation involves the following steps.

1. Compute the grand mean score across all speaker–listener combinations. This is 49% correct in the present example (see bottom row).
2. Next, compute the mean score for each of the speaker groups (by averaging over the listener groups). For instance, the mean score for Chinese speakers is 39 (see column speaker-mean), which is the mean of Chinese speakers combined with Chinese, Dutch, and American listeners, with scores of 30%, 40%, and 50%, respectively (see column Obs.).^1^
3. Likewise, compute the mean scores for each of the listener groups, averaged over speakers. This yields mean scores of 33%, 53%, and 61% for Chinese, Dutch, and American listeners, respectively (see column listener-mean).
4. Then compute the deviation of the speaker means from the grand mean by subtraction. For instance, the mean of the Chinese speaker group (39%) is 10 points below the grand mean of 49%, hence a deviation of −10 (see column speaker-effect).
5. Similarly, compute the deviation of each listener group mean from the grand mean. The mean of the Chinese listener group (33%) is 16 points below the grand mean, hence a deviation of −16 (see column listener-effect).
6. Then compute the expected score for each speaker–listener combination, by adding the speaker group deviation and the listener group deviation to the grand mean. In the case of the Chinese-Chinese speaker-listener combination this would be 49% (grand mean)−16 (listener group deviation)−10 (speaker group deviation) = 22% (see column Exp.).^2^
7. Finally, compute the prediction error (“residual”) for each speaker–listener combination, which is the difference between the expected and the observed score. This gap between expected and observed scores is then closed by the interaction component in the linear model. For the Chinese–Chinese combination, we expect 22% (top data row, column Exp.) but find 30% (same row, column Obs.), so that the residual equals +8 points (same row, column Δ). This is the value for R-ISIB.^3^ Note that the mean R-ISIB for each row and each column in the matrix, as well as for the matrix in its entirety, should always add up to zero, since positive and negative prediction errors should cancel each other out.

When the listeners are Chinese, Dutch, and American, the expected mean scores are −16, +4, and +12 relative to the grand mean; for the three speaker language backgrounds, the expected mean should be additionally corrected with −10, +3, and +7, respectively. Note here that the size of the increments/decrements is larger for listener language background than for speaker language background, that is, the listener effect is larger than the speaker effect.

Generally, the observed scores are correctly predicted or even overestimated by the linear addition of the two main effects. Only in three combinations of factor levels is the observed score substantially better than the prediction. These are precisely the conditions in which the listeners are confronted with vowel tokens spoken by their fellow countrymen (“shared interlanguage”, shaded rows in Table 2). The native or interlanguage benefit is 4 to 8 percentage points better than the expected score. It appears that there is no need to differentiate between communication between a native speaker and a native listener (with a R-ISIB of +7 points, which could be called a “native-language benefit”) and communication between a nonnative speaker and a nonnative listener who share the same native language (+4 and +8 points for Dutch and Chinese matched interlanguage groups, respectively): in both situations the residual is of comparable, positive magnitude.

In the case of a speaker–listener combination with a mixed interlanguage, the R-ISIB is very close to zero: −1 for Dutch-Chinese and −2 for Chinese-Dutch). This would indicate that, indeed, the shared interlanguage yields a substantially greater benefit than the mixed interlanguage. There are too few observations to run any meaningful statistics on the difference; this we will do in the next section of this article where we will test this effect on data aggregated over a number of studies.

R-ISIB is most negative when the speaker–listener combination involves one native and one nonnative party. Here the R-ISIB ranges between −1 and −6 points. Again, we will defer statistical testing of the significance of this native-language handicap until we have sufficient aggregate data.

In the next section, we will first provide some background on the idea of linear modeling and then explain the computational procedure that should be applied to compute the proposed relative measure of interlanguage benefit. Here we will use an example taken from Wang (2007). In the later sections, we will reanalyze earlier results by Smith and Rafiqzad (1979), by Bent and Bradlow (2003) and the set of six tests used by Wang (2007). As far as we have been able to ascertain, these are the only studies that have been published on mutual intelligibility in English comparing speaker and listener groups from more than just two (i.e., native English vs. one nonnative language group) different nonnative language backgrounds.

## Meta-Analysis: Testing the ISIB Hypotheses on Aggregated Data

In this section, we will test the restricted (shared interlanguage) and generalized (mixed interlanguage) versions of the ISIB hypothesis on a collection of data taken from three studies that address the issue. Where individual studies contain too few data to do any meaningful statistical evaluation of the versions of the hypotheses, we claim that aggregate data from multiple studies do afford this possibility. In the next subsection, we will briefly describe the studies that underlie the meta-analysis, then formulate quantitative predictions and proceed with a statistical analysis of the aggregate data.

### Literature Data

#### Smith and Rafiqzad (1979)

The earliest study to compare the intelligibility of native and nonnative English in a sufficiently complete matrix of speaker and listener groups with a variety of language backgrounds was probably done by Smith and Rafiqzad (1979). In this study, the authors recorded materials from educated L2 speakers of English in seven Asian countries, viz. Hong Kong, India, Japan, Korea, Malaysia, Nepal, and the Philippines. Similar materials were collected from native speakers of American English. Unfortunately, the design was incomplete in that no materials of any speaker group were presented to American native listeners. There were also nonnative listener groups that were never used as speakers — these I pruned from the matrix below.^4^ The materials were presented to the seven relevant listener groups in a Cloze test, in which listeners saw a printed version of the audible text, with every sixth word replaced by a blank to be filled in. A detailed analysis of the results both in terms of ISIB and of R-ISIB, as was demonstrated for the Wang’s (2007) vowel identification experiment in A Relative R-ISIB section, is presented in Van Heuven (2015; Tables 2 and 3). Here we will not dwell on the intermediate steps but concentrate on a comparison of effects across all meta-analytic data.

**Table 3.** Mean (Absolute) ISIB and R-ISIB Broken Down by Four Types of Speaker–Listener Group Combinations, Aggregated Over all Experiments Reviewed in This Article.

Speaker–listener combination	Absolute (ISIB)			Relative (R-ISIB)		
	Mean	*SD*	*N*	Mean	*SD*	*N*
1. Shared interlanguage	63.1	21.9	26	6.8	8.5	26
2. Mixed interlanguage	59.4	24.7	62	−2.4	6.1	62
3. One native	63.8	21.5	35	−2.3	5.3	35
4. All native	92.7	10.7	7	7.0	7.7	7
ANOVA	*F*(3,126) = 4.5, *p* = .005, η^2 ^= .096			*F*(3,126) = 16.2, *p* << .001, η^2 ^= .279		
Posthoc (Bonferroni, α = .05)	{2, 1, 3} < {4}			{3, 2} < {1, 4}		

ISIB = interlanguage speech intelligibility benefit; R-ISIB = relative-interlanguage speech intelligibility benefit; ANOVA = analysis of variance.

#### Bent and Bradlow (2003)

Bent and Bradlow (2003) examined the interlanguage benefit in a database with mutual intelligibility scores in English obtained for five types of speakers: one high-proficiency and one low-proficiency Korean L2 speaker of English, one high-proficiency and one low-proficiency Chinese L2 speaker of English, and one native speaker of American English. Sentences produced by these five (female) speakers were presented to four groups of listeners with Chinese (*N* = 21), Korean (*N* = 10), American (*N* = 21), and mixed-foreign (*N* = 12) backgrounds. Intelligibility scores were determined for all 5 × 4 = 20 combinations of speaker and hearer L1 backgrounds.

The scores were not expressed in percentages but in rationalized arcsine units (RAUs). The arcsine transform was applied by Bent and Bradlow to unwarp the bottom and top ranges of the percentage scale in order to compensate for bottom and ceiling effects. After “rationalisation” the transformed scale extends between −17 and +117 RAU; 50 RAU = 50% (Studebaker, 1985). For the purpose of this meta-analysis, we will treat the RAU scores as if they were percentages. This decision is motivated by the consideration that whatever distortion the arcsine transformation may have introduced, the choice between percentages and RAUs will not affect the difference between absolute and relative ISIB. For details of the results, see Van Heuven (2015: Tables 4 and 5). The absolute and relative (R-ISIB) scores were transferred to the aggregate data.

#### Wang (2007)

Wang (2007) ran a large study on the mutual intelligibility of Dutch, Mandarin, and American speakers of English. Twenty speakers (10 males, 10 females) from each of these three different native-language backgrounds produced materials in English, that is, (a) vowels in a /hVd/ context, (b) consonants and (c) consonant clusters in intervocalic contexts, (d) semantically unpredictable sentences, (e) semantically meaningful sentences with final target words in unpredictable (“non-pregnant”), and (f) predictable (“pregnant”) contexts. The materials of one representative male and one female speaker for each of the three language backgrounds were then offered for identification (of vowels, consonants, and clusters) or recognition (of words in sentences) to 36 listeners in each of three countries 36 learners of English at Changchun University (Mandarin language area), 36 learners of English at Leiden University (Netherlands), and 36 American native listeners at the University of California Los Angeles (UCLA), so that all nine possible combinations of speaker and listener backgrounds occurred equally often in the experiment.

### Predictions

We will now perform a statistical analysis across all data that were discussed above. We will specifically test two related hypotheses. The first is that (1a) there will be a strong interlanguage intelligibility benefit such that two nonnatives with the same mother tongue will understand each other in English best when speaking a foreign language (shared interlanguage), (1b) two nonnatives with different native language backgrounds will understand each other more poorly (mixed interlanguage), and (1c) the poorest intelligibility will be observed when a nonnative communicates with a native speaker (whether as speaker or as listener). The second hypothesis is that these predictions will be borne out more clearly when using the relative measure of the ISIB than when looking at absolute intelligibility scores.

### Results of the Meta-Analysis

The aggregate data contain 130 cases, that is, the total number of speaker–listener group combinations accumulated across the studies reviewed in the preceding subsections, that is, 7 (listener language backgrounds) × 8 (speaker language backgrounds) = 56 combinations taken from Smith and Rafiqzad (1979), 4 × 5 = 20 combinations from Bent and Bradlow (2003), and 6 (tests) × 3 (listener groups) × 3 (speaker groups) = 54 combinations from Wang (2007). The six tests from Wang (2007) were treated as independent, on the strength of the observation that there were no significant correlations between the scores obtained on the six tests (see Wang 2007, chap. 10). Table 3 presents the mean ISIB and R-ISIB values for four types of speaker–listener group combinations, that is, combinations yielding (a) shared interlanguage, (b) mixed interlanguage, (c) native/nonnative pair, and (d) native-native pairs (as a control condition).

In terms of absolute interlanguage benefit, the results indicate that native-speaker-native-listener pairs yield near-ceiling intelligibility scores (93%), which is about 30 percentage points better than any of the three combinations involving one or two nonnative interactants; these three speaker–listener combinations do not differ from each other by a posthoc comparison of means (Bonferroni-corrected, after one-way analysis of variance, see Table 3). The results obviously contradict the hypothesis that there is any benefit to be gained by nonnatives, whether they do or do not share an interlanguage: all nonnatives are equally handicapped, whether communicating with a native or with each other.

In relative terms, however, the situation is much more as predicted. First of all, nonnatives with a shared interlanguage enjoy the same intelligibility benefit as two natives, with positive R-ISIB values of 6.8 and 7.0, respectively. Moreover, when speaker and listener have a non-matched (mixed) interlanguage, the R-ISIB is negative (−2.4). There is a clear difference, then, between the matched and the non-matched interlanguage pairs to the effect that no benefit remains when speaker and listener have different native languages. The idea of a native speaker handicap, however, is not supported by the aggregate data. It is not the case that a nonnative listener is at a greater disadvantage when communicating with a native speaker than when communicating with a nonnative with whom he does not share the native language background. Not only are the R-ISIB results more in line with the hypotheses formulated in the literature, they are also statistically more reliable, given that the effect size (η^2^) of the speaker–listener combination is roughly three times stronger in relative (R-ISIB) than absolute (ISIB) scores (see Table 2).

## Conclusion and Discussion

On the basis of the literature, we formulated two hypotheses with respect to the effect of the specific composition of a speaker-listener pair involving different combinations of native and nonnative interactants. The first hypothesis predicted (1a) that two nonnatives will understand each other in English best when they have the same native-language background (i.e., shared interlanguage) and (1b) will perform better than when they have different native language backgrounds (mixed interlanguage). These subhypotheses proved false when tested in absolute terms but were clearly supported by the data when evaluated in relative (R-ISIB) terms. In fact, in relative terms, nonnative speaker-listener pairs enjoy the same interlanguage benefit as native speaker-native listener pairs — as long as speaker and listener have the same mother tongue. One more subhypothesis, (1c), which was formulated on the basis of earlier analyses of Wang’s (2007) data, cannot be upheld in the meta-analysis: it is not the case that communication between native and nonnative interactants is poorer than between two nonnatives with different language backgrounds. On the strength of this latter finding, hypothesis (1c) has to be rejected.

The above state of affairs suggests that the earlier tripartite division of interlocuters in terms of (a) native speakers and listeners, (b) nonnative speaker-listener pairs with a shared interlanguage, and (c) those with non-shared, that is, mixed, interlanguages is not supported by our R-ISIB results. The meta-analysis boils down to a very simple and clear-cut binary division in intelligibility between native and nonnative speakers of a language. When two interactants share the same native language, they enjoy the advantage of a shared phonology. In this sense native speakers communicating with native listeners also share a common interlanguage, namely the ideal (near) perfect grammar/phonology of the native speaker/listener. When two interactants do not have the same mother tongue, their mutual intelligibility is poorer. Here, it does not matter whether both interactants are nonnative or whether a foreigner communicates with a native — the point is that they do not share any interlanguage.

Also Hypothesis (2) predicted that the effects would be stronger when evaluated in relative rather than in absolute scores. The analysis of variance indicated an effect size in R-ISIB that was three times larger than when analyzed in absolute scores. Hypothesis (2) is therefore confirmed: relative scores work better than absolute scores.

As we pointed out in the introduction, it has been observed that nonnative listeners of English often have the intuition that they understand a fellow nonnative talker, that is, one with whom they share a common mother tongue, better than a native speaker of English. This intuition is supported by the experimental data, but only when the intelligibility scores are expressed in relative terms, that is, in terms of the R-ISIB measure that was explained in A Relative R-ISIB section. We conclude, therefore, that the proper way of evaluating the concept of the ISIB, as formulated by Bent and Bradlow (2003), is in relative rather than in absolute terms. We would suggest that future research on communication between native and/or nonnative interactants should analyze the results in terms of our R-ISIB measure, either as the only ISIB measure or at least as a measure supplementary to comparing absolute ISIB values as has been the common practice in the literature.

In summary, we now have at our disposal a way to quantify the ISIB in a way that works and that matches intuitions that were formulated in the literature (which were not matched when absolute intelligibility scores were examined earlier). Also, we now know that there is a clear (and motivated) difference between communication that involves interactants who share the same native language (whether speaking in their mother tongue or in a nonnative language) and communication involving interactants who do not share the interlanguage. This refutes the earlier hypothesis (entertained and not rejected by Bent & Bradlow, 2003) that any nonnative speaker should be more intelligible to a nonnative listener than a native speaker is. This insight is new. Together these two points constitute the contribution this article adds to what was known about factors that determine speech intelligibility among native and nonnative interlocutors.

## Declaration of Conflicting Interests

The author(s) declared no potential conflicts of interest with respect to the research, authorship, and/or publication of this article.

## Funding

The author(s) disclosed receipt of the following financial support for the research, authorship, and/or publication of this article: The authors acknowledge the financial support by the Ministry of Education, Humanities and Social Sciences Planning Project, 14YJA740036; The Department of Education of Guangdong Province Project 11.5 on Educational Science 2011TJK357; Shenzhen Science and Technology Innovation Committee Basic Research Project, 007570.

## Notes

1 On face value, these three numbers should average out at 40% instead of 39%. The discrepancy is due to rounding errors.

2 On the basis of the values presented in Table 1, an expected value of 21% would be expected. The slight discrepancy is due to greater rounding accuracy in the computations underlying the table.

3 The numbers presented in this table deviate slightly from what was published in Wang (2007) and Van Heuven and Wang (2007). The present numbers are correct.

4 Smith and Rafiqzad (1979) have been criticized for other reasons as well. It has been pointed out that the materials produced by the speaker groups differ substantially in terms of conceptual comprehensibility — so that no straightforward comparisons between speaker and listener groups can be made. This criticism, of course, is no longer valid once we apply the concept of R-ISIB. When the speaker of a language is more difficult to understand, for whatever reason, this will affect the main effect of speaker but not the speaker by listener interaction, that is, not the R-ISIB.
